# Visualizing hypoxic modulation of beta cell secretions via a sensor augmented oxygen gradient

**DOI:** 10.1038/s41378-022-00482-z

**Published:** 2023-02-07

**Authors:** Kai Duan, Mengyang Zhou, Yong Wang, Jose Oberholzer, Joe F. Lo

**Affiliations:** 1 Department of Mechanical Engineering, Bioengineering Program, University of Michigan at Dearborn, Dearborn, MI 48128 USA; 2 Department of Surgery/Transplant, University of Virginia, Charlottesville, VA 22908 USA

**Keywords:** Microfluidics, Sensors

## Abstract

One distinct advantage of microfluidic-based cell assays is their scalability for multiple concentrations or gradients. Microfluidic scaling can be extremely powerful when combining multiple parameters and modalities. Moreover, in situ stimulation and detection eliminates variability between individual bioassays. However, conventional microfluidics must combat diffusion, which limits the spatial distance and time for molecules traveling through microchannels. Here, we leveraged a multilayered microfluidic approach to integrate a novel oxygen gradient (0–20%) with an enhanced hydrogel sensor to study pancreatic beta cells. This enabled our microfluidics to achieve spatiotemporal detection that is difficult to achieve with traditional microfluidics. Using this device, we demonstrated the in situ detection of calcium, insulin, and ATP (adenosine triphosphate) in response to glucose and oxygen stimulation. Specifically, insulin was quantified at levels as low as 25 pg/mL using our imaging technique. Furthermore, by analyzing the spatial detection data dynamically over time, we uncovered a new relationship between oxygen and beta cell oscillations. We observed an optimum oxygen level between 10 and 12%, which is neither hypoxic nor normoxic in the conventional cell culture sense. These results provide evidence to support the current islet oscillator model. In future applications, this spatial microfluidic technique can be adapted for discrete protein detection in a robust platform to study numerous oxygen-dependent tissue dysfunctions.

## Graphical abstract

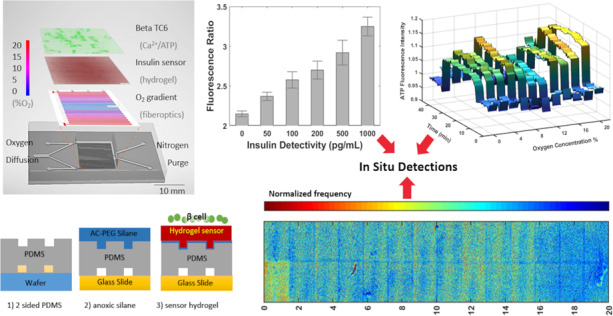

## Introduction

Spatial transport and concentrations of molecules between cells are important in many physiological processes. Specifically, oxygen, calcium, insulin, and other signaling molecules play major roles in diabetes-related pathologies. Oxygen and hypoxia, for instance, are closely controlled via hypoxia-inducible factors and exhibit spatial gradients in diabetic tissues (fat and islets)^1–13^. Furthermore, intracellular calcium and cell-to-cell calcium transport demonstrate dynamic concentrations in pancreatic beta cells^14–16^. However, contemporary techniques using molecular assays and fluorescence microscopy can only determine responses at single concentrations and static time points, rather than a gradient of concentrations dynamically over time. These assays and microscopy techniques were designed for discrete concentrations and require lengthy sample preparations. To address these limitations, microfluidic devices have been realized to enable spatial and dynamic studies of molecular transport in islets and beta cells, providing rapid transient detection or microscopy imaging of pathophysiological conditions in diabetes^17–21^. However, these contemporary microfluidics prioritize either spatial gradients or fast detection dynamics as they combat diffusion in microchannels. We previously addressed this diffusion limitation by coupling nitrogen convection to drive diffusion^22^. In this work, we further leveraged our spatial microfluidic oxygen gradient combined with integrated calcium, ATP, and insulin detection to study beta cell hypoxia in situ. In contrast to previous hypoxia studies^18^, in this study, a gradient of oxygen concentrations was applied across an entire Beta TC6 culture. Thus, we extended current detection techniques in both spatial and temporal dimensions for beta cell studies. Using this novel technique, we uncovered a relationship between calcium and insulin modulation as a function of oxygen levels.

### Islet calcium and insulin oscillations

To illustrate the dynamics of signaling molecules in beta cells, we summarize here the process of the glucose-stimulated insulin response (GSIR). GSIR begins with glucose uptake, followed by an increase in the ATP/ADP (adenosine triphosphate/adenosine diphosphate) ratio due to glucose metabolism. This upsurge in ATP closes ATP-dependent potassium (K_APT_) channels, which depolarizes the membrane and triggers a calcium influx through voltage-dependent calcium channels (VDCCs). This intracellular calcium flux then prompts the exocytosis of packaged insulin vesicles. In vitro and in vivo, both calcium flux and insulin secretions exhibit oscillations on a time scale from 10 seconds to several minutes^14–16,23–25^. These calcium oscillations propagate as a wave across beta cells in an islet^14–21,23–26^. The faster oscillations are based on electrical modulations of membrane potentials^23,27,28^, while the slower, minute-scaled oscillations are based on metabolite modulations^23,28,29^. Oxygen can modulate membrane channels responsible for electrical oscillations^18,30–32^. Oxygen can also modify glucose metabolism and thus affect metabolite oscillations^23,30,33,34^. These aspects of calcium oscillation represent cell-to-cell communications in islets^14–16,26^, of which oxygen may be a key modulator. While both types of oscillation affect insulin secretion^23,35^, mitochondrial ATP specifically affects GSIR sensitivity^15,36^. However, a systematic assay of oxygen on calcium or insulin using a gradient is lacking. To visualize these temporal oscillations and their modulation by oxygen, a spatial gradient with integrated GSIR detection would be the ideal experimental platform. Moreover, such a platform could test whether Beta TC6 cells can mount the same kind of ATP responses to glucose stimulation as other beta cell lines (see supplementary data) and whether their ATP responses coincide with calcium or insulin modulation across the cell culture.

### Advantages of gradient-based multimodal microfluidics

**Fig. 1:**
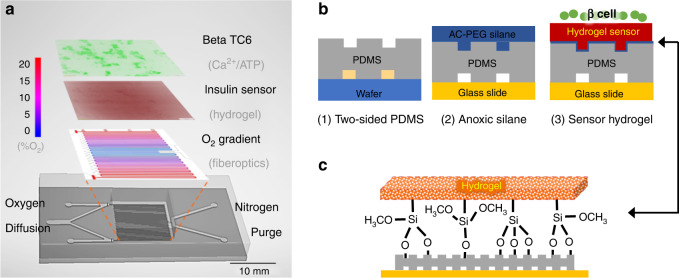
Microfluidic oxygen gradient with spatial hydrogel sensor. **a** The microfluidic device contains multiple layers. The gas layer balances oxygen diffusion (red) with nitrogen convection (blue) to create a 0–20% gradient as measured with a fiber optic oxygen probe. Note that the gradient is symmetrical, so either half can be used. The 200 µM polydimethylsiloxane (PDMS) membrane that defines the gas channels is highly permeable to oxygen and diffuses the gradient to the hydrogel and cell culture seeded atop. On top of the oxygen gradient, a porous hydrogel sensor was bonded to the microchannels. This hydrogel encapsulates anti-insulin capture antibodies, 20k polyethylene glycol (PEG) porogen, and arginylglycylaspartic acid (RGD) peptides to provide cell attachment and insulin detection. The open top aqueous reservoir allows normal cell culture processes and fluorescence microscopy. **b** The fabrication process of the multilayered microfluidics begins with normal photolithography and PDMS molding. During the chemical surface modifications, a nitrogen chamber is used to prevent oxygen inhibition of hydrogel cross-linking at the PDMS–gel interface. The resultant sandwich is washed with buffer to remove uncross-linked reagents and porogens to complete the porosity-enhanced hydrogel sensor. **c** Moreover, attachment of the functionalized hydrogel is achieved through both topological and chemical surface modifications. Microtextures are patterned on the cell facing the side of the PDMS membrane to increase the surface area in contact. Then, the sandwich is incubated with acrylated silane to create Si–O-bonds to tether the hydrogel backbones

To enable oxygen modulation and spatial detection within the same device, we designed a multilayered construct by integrating our membrane-based gas microfluidics^18,19,22^ with a porous hydrogel sensor layer^37,38^ (Fig. 1). As described in our previous work, this gas microfluidics balanced oxygen diffusion against nitrogen convection^22^ and expressed a 0-20% hypoxia gradient across a 200 µM polydimethylsiloxane (PDMS) membrane (Fig. 1a). Note that the gradient was designed to span the range below atmospheric oxygen (21%) with symmetry so that both halves could be used. Across this membrane, a polyethyl glycol (PEG)-based hydrogel was created to provide a surface for cell attachment and insulin immunodetection. Cell attachment was enabled via Arginylglycylaspartic acid (RGD) peptides copolymerized in the hydrogel. Insulin detection provided capture antibody within the hydrogel. Additionally, porosity generated via nonacrylated PEG fragments enhanced the molecular transport and thus detection sensitivity in the hydrogel^37,38^. The complete microfluidic device was fabricated by bonding the multiple layers one by one, as shown in Fig. 1b. To physically immobilize this multifunctional hydrogel, both silane chemistry and surface texturization were applied to enhance gel–PDMS bonding (Fig. 1c).

As a result, the device was capable of dynamically imaging GSIR with in situ calcium, ATP, and insulin detection. Together with the integrated oxygen gradient, the device increased the throughput of conducting beta cell experiments. Our design utilized both enhancements in scaling and reaction kinetics afforded by microfluidics. It offered novel visualization of calcium oscillations across beta cell cultures, a multiplexing of spatial and temporal parameters that was difficult to achieve using contemporary microfluidics.

## Results

### Surface modifications improved hydrogel adhesion

**Fig. 2:**
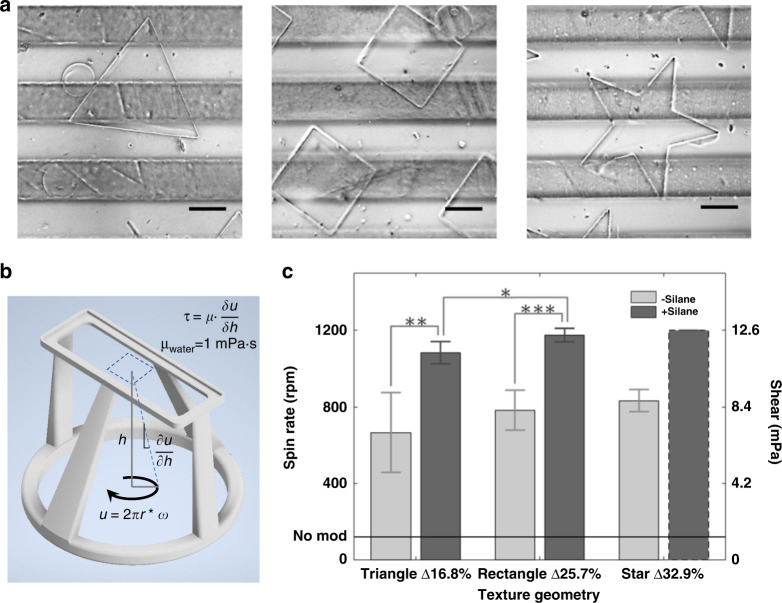
Hydrogel adhesion characterized via fluidic shearing. **a** Microtextures were employed to increase the bonding areas in addition to chemical surface modifications. From left to right, triangular, square, and star-shaped dimples were micropatterned at 100 µm depths on the PDMS membrane atop the gas microchannels. The increase in the number of sides of these microstructures increased the cross-sectional contact areas by 16.8%, 25.7%, and 32.9%, respectively, resulting in stronger hydrogel bonding (scale bars denote 200 µm). **b** To quantify the improved hydrogel adhesion, a fluidic shearing apparatus was designed and 3D printed in polylactic acid (PLA). The apparatus positioned hydrogel integrated devices upside down in a beaker of water, exposing the gel to rotational shear produced by a stir bar atop a stir plate. The angular rate of rotation translated to the linear speed at the edge of the hydrogel can be projected to the gel surface to define a shear stress *τ*. When the shear stress reaches the limit of gel adhesion, delamination is observed, and the rotational rate and shear stress are recorded. **c** The unmodified hydrogel (“no mod”) provided a baseline for comparison to enhanced bonding. Significant increases were found when both silane and microtexture were used together, with rectangular and star shapes topping the measurable stress at 12.6 mPa. Please note that the star-shaped membrane exceeded the stir plate speed of 1200 rpm and never showed delamination. Increases in cross-sectional area as percentages were noted for each shape. *p < 0.05, **p < 0.01, and ***p < 0.001

Although the hydrogel biosensor was not exposed to external mechanical forces, it acted as a substrate for cell seeding and was subject to handling and fluid exchanges during cell culturing. Direct placement of the gel on PDMS resulted in gel delamination and lift-off from the surfaces, causing uneven spots in the spatial biosensing. To address these issues, both chemical and topological—i.e., silanization and texturization—modifications were employed: PEG chains were attached directly to the exposed PDMS hydroxyl groups via silanization. Microtexture dimples with increasing numbers of sides—triangle, square, and star—were patterned. These increased the cross-sectional bonding surface by 16.8%, 25.7%, and 32.9%, respectively (Fig. 2a). Both modifications contributed to the strength of adhesion between the hydrogel and PDMS.

To quantify the adhesion, a fluidic shear apparatus was devised via 3D printing. The apparatus was submerged in water, and a standard stir plate was employed to create rotational shear against the hydrogel mounted inversely atop the apparatus (Fig. 2b). At a specified angular rotation ω, the fluid velocity *u* at the projected perimeter of the hydrogel was calculated. This velocity decreased toward the hydrogel surface, where the no-slip condition was assumed. With the slope of this velocity profile, $\frac{{\partial u}}{{\partial h}}$, and a given viscosity, e.g., *µ*_water_, the shear stress experienced by the hydrogel edge was calculated. The angular rotation ω at which the hydrogel delaminated was recorded for unmodified hydrogel, silanized hydrogel, textured hydrogel, and texture + silanized hydrogel preparations (Fig. 2c). Silanization alone improved the attachment over unmodified hydrogel (baseline at 125 rpm or 1.3 mPa). Texturization increased hydrogel adhesion across geometries. Moreover, texture + silanized hydrogels showed a significant increase over texturization alone, with additional increases from triangle to square textures. With the star geometry, no delamination was observed at the highest stir plate rotation (1200 rpm), suggesting that even greater shear stress could be tolerated. Hence, silane-modified, star-textured hydrogel bonding was selected for all subsequent experiments.

### Optimized porous hydrogel spatial insulin detection

**Fig. 3:**
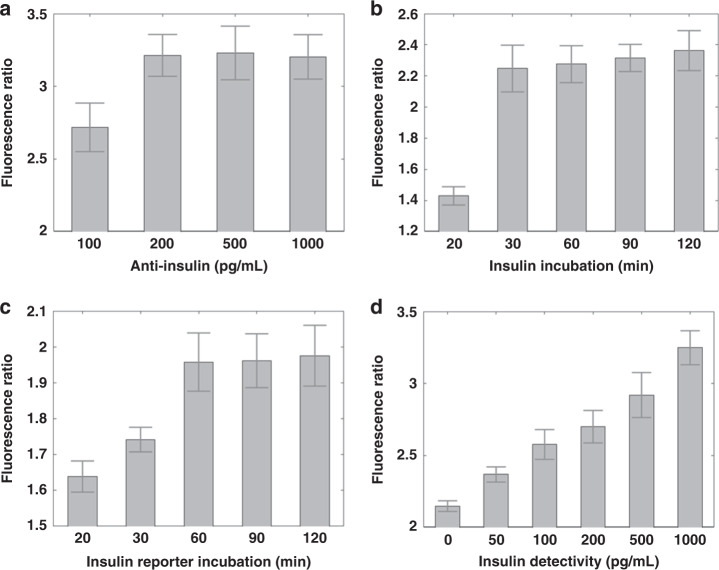
The optimized spatial insulin sensor achieved a 25 pg/mL limit of detection. The assay was optimized by using the fluorescence ratio as the readout, where the signal intensity was divided by the background fluorescence in the hydrogel. **a** The amount of capture antibody was tested in the porous hydrogel by varying the concentrations from 100 to 1000 pg/mL. Treatment with capture antibody at excess of 200 pg/mL resulted in no observed significant increase in the fluorescence ratio. **b** Next, the incubation time for target insulin was tested from 20 to 120 min. The incubation time was markedly improved compared to that of conventional assays at 30 min. **c** Then, the reporter antibody incubation was tested, and the optimal time was 60 min. **d** Finally, a detectivity curve from 50 to 1000 pg/mL was assayed to determine the limit of detection. Using the convention of twice the standard deviation of the blank sample, the insulin limit of detection in our spatial sensor was 25 pg/mL

The 100 µm PEG hydrogel covered the entire 1.5 × 1.5 cm cell culture area to provide spatial detection of insulin secretion on top of the oxygen gradient. The hydrogel provided enhanced by porosity to enhance molecular transport^37,38^. The integrated insulin immunodetection was optimized for assay speed and sensitivity. By measuring the fluorescence ratio, i.e., signal to background fluorescence, the optimal capture antibody concentration was found to be 200 pg/mL (Fig. 3a). The target insulin and final reporter incubation times were optimized at 30 and 60 min, respectively, beyond which no further improvements were seen (Fig. 3b, c). Profiling concentrations from 50-1000 pg/mL resulted in the detectivity curve shown in Fig. 3d, with a calculated limit of detection of approximately 25 pg/mL. This limit of detection is half of what is typical for insulin ELISA kits (~50 pg/mL) yet allows spatial readouts in tandem with the oxygen gradient. This improvement is typical of what we achieved using porous hydrogels for other biomolecular detection methods.

### Simultaneous detection of calcium and insulin in beta TC6

**Fig. 4:**
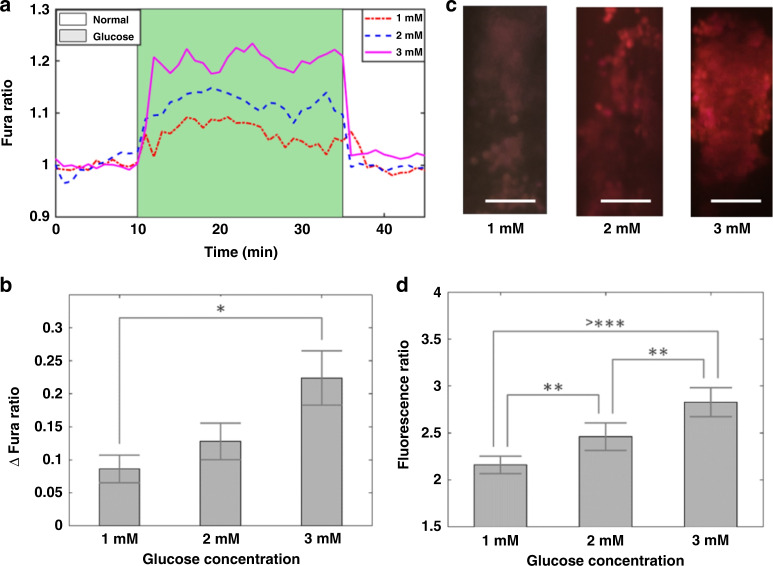
Simultaneous detection of calcium and insulin in beta TC6 cells. The microfluidic platform was first tested for GSIR under normoxic conditions at a single location without spatial oxygen gradients. Boluses of 1–3 mM glucose were injected into the aqueous reservoir during observation via microscopy for 25 min before flushing with Krebs–Ringer buffer to return to baseline. Intracellular calcium was monitored using Fura-2 AM dye, while insulin release was detected using the hydrogel assay. **a** Beta TC6 cells responded to glucose stimuli and demonstrated intracellular calcium flux, with 3 mM resulting in the highest Fura measurements. Here, Fura was sampled at 15 s intervals. At this sampling rate, cyclic oscillation was observed at the 3 mM concentration. However, at lower concentrations, the noise averaged out the oscillations. **b** The change in the Fura ratio from baseline to peak was plotted for the three concentrations. Significant increases were seen in 3 mM over 1 mM stimulation. **c** Corresponding fluorescence micrographs on the right show increased insulin detection with increasing glucose stimulation (scale bar denotes 100 µm). **d** Insulin release detected using the built-in sensor demonstrated significant increases at all concentrations. *p < 0.05, **p < 0.01, and ***p < 0.001

We quantified beta cell calcium and insulin responses to glucose stimulation under normoxic conditions using Fura-2 and the integrated hydrogel sensor. Beta TC6 cells were cultured on the surface of the 100 µm sensor hydrogel. The RGD group copolymerized in the hydrogel enabled the attachment of beta cells (see S1). After culturing beta cells without glucose for 24 h, we stimulated them using Krebs–Ringer buffer spiked with different glucose concentrations: 1, 2, and 3 mM^39^. The Fura-2 ratio of 340–380 nm intensities increased with increasing glucose concentration from 1 to 3 mM. Here, Fura signals were sampled at 15 s intervals, showing cyclic oscillations at 3 mM stimulation (Fig. 4a). The 3 mM glucose group also exhibited a significantly higher Fura ratio than the 1 mM glucose group (Fig. 4b). Using the integrated hydrogel sensor, we measured 2.36 pg/mL insulin for the 1 mM glucose stimulation, 72.0 pg/mL insulin for the 2 mM glucose stimulation, and 373 pg/mL insulin for the 3 mM glucose stimulation (Fig. 4c). The insulin response showed a linear increase with higher glucose concentrations (Fig. 4d). As the 3 mM stimulation exhibited the most robust calcium and insulin responses, all subsequent experiments were performed using this glucose concentration.

### Spatial gradient with in situ calcium, insulin, and ATP detection

**Fig. 5:**
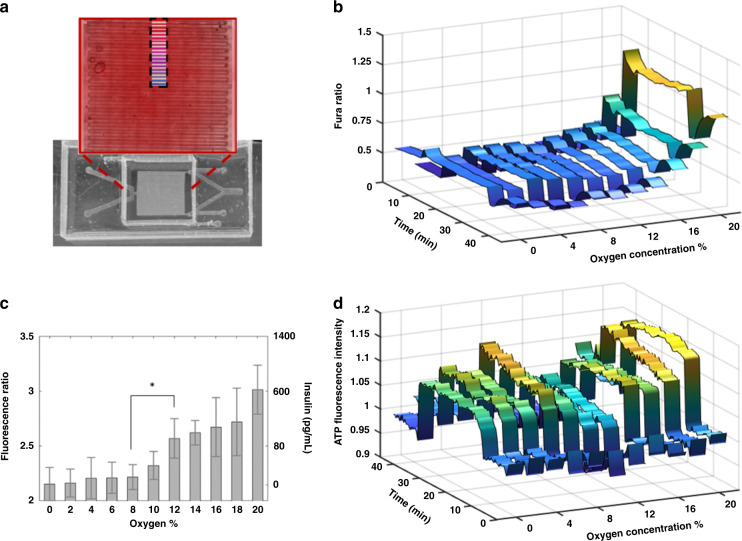
Oxygen gradient stimulation with spatiotemporal detection. **a** Eleven positions within the oxygen gradient were selected as regions of interest for calcium, ATP, and insulin responses. **b** Fura ratios for oxygen concentrations were recorded at 1-min intervals for 46 time points. The Fura ratios remained relatively low until above 10%, where they increased with increasing oxygen. The observed transient oscillations suggested nuances to calcium signaling, which led to subsequent Fourier analysis. Note that this 1-min sampling was significantly slower than the 15 s acquisition that we achieved for the fixed location, single oxygen imaging in the previous figure. Thus, these results could potentially underestimate actual calcium oscillations. **c** Insulin secretion was quantified via a built-in sensor. A jump occurred at oxygen levels of approximately 8–12% (*p = 0.057). **d** Mitochondrial ATP was quantified via fluorescence labeling, showing modest increases until the same 8–12% oxygen range, where the response dipped before rising again near 20%. These results suggested that Beta TC6 cells did mount ATP responses to glucose. These responses exhibited a transition between 8–12% oxygen that may be related to changes in metabolites

To study the effect of different oxygen concentrations on GSIR, we applied microfluidic spatial oxygen gradients to Beta TC6 cells. Leveraging the device’s symmetry, we acquired half of the gradient using a 2 × 12 frame microscopy collage at 10× magnification and then analyzed eleven positions within the 0–20% oxygen range (Fig. 5a). Calcium flux and insulin release were quantified at 1-min time intervals for 46 min. In response to 3 mM glucose boluses, the Fura ratio difference did not change much between 0 and 10% oxygen concentrations. For concentrations higher than 10%, the ratio increased with increasing oxygen concentration (Fig. 5b). From 0 to 10% oxygen, insulin secretion increased slowly from 1.17 to 39.8 pg/mL. At 12% oxygen, however, insulin secretion jumped significantly, *p* = 0.057, and subsequently rose from 98.31 to 641.68 pg/mL from 12 to 20%, Fig. 5c. These results were consistent with our previous findings of a significant impairment of GSIR in whole islets under 10% oxygen^12,13^. In addition, the mitochondrial ATP response rose between 0 and 6% oxygen concentrations and then dipped in the same 8–12% oxygen range, followed by a recovery toward 20% oxygen (Fig. 5d). The results showed that Beta TC6 cells did mount a modest ATP response in the presence of glucose stimulation compared to other beta cell lines (see Supplementary Data S2). The results also suggested that the changes from 8 to 12% are related to metabolic activities, with no visible changes to the static magnitudes of calcium responses. This prompted our investigations of calcium oscillations using Fourier analysis (see the next section).

### Oxygen modulation of calcium dynamics revealed by spatiotemporal imaging

**Fig. 6:**
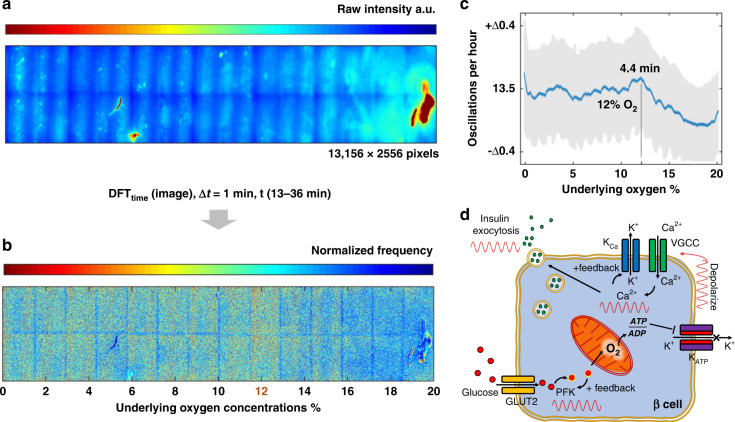
The specific oxygen level increased the frequency of calcium oscillations. A section of the symmetric oxygen gradient was acquired using stage scanning fluorescence microscopy, encompassing a 0–20% gradient as characterized earlier. **a** The resultant raw intensity image has 13,156 × 2556 pixels from the collage, or 33 Megapixels total per image. Artifacts visible in these images were removed prior to analysis. Collage images were acquired at 1-min intervals for 46 min. Only time points corresponding to glucose boluses were used, giving 24 points from 13 to 36 min. The initial overshoot at 12 min was removed to reduce artifacts (see Supplementary Data S3). **b** A per-pixel time-based Fourier transform was applied, and the magnitude profile was weighed at the center of gravity, giving a nominal frequency value for each pixel. The result was replotted to show this frequency versus underlying oxygen concentrations, where an intense region approximately 12% oxygen was observed (visible banding seen in collage was due to image stitching). **c** The pixels were averaged and flattened vertically to show a frequency profile versus oxygen concentrations. The resultant profile showed a peak oscillation at 12% oxygen. This oxygen level is neither hypoxic nor normoxic in the standard cell culture sense. **d** A conventional dual oscillator model for calcium includes both electrical and metabolic components. Feedback in the PFK portion of glycolysis is viewed as the center of metabolic oscillation, whereas calcium channel feedback is viewed as the center for electrical oscillations. Oxygen, due to its effect on the mitochondria, could be a key factor in both oscillator components

A difference in calcium oscillation was observed near the 8–10% range of the oxygen gradient (Fig. 5b). To further quantify this change in calcium dynamics, frequency analysis was applied to the series of time-lapse images. To reduce the data size and the time of calculation, a 33 megapixel area over 24 time points (duration of GSIR pulse) was used for analysis (Fig. 6a). A Fourier transform was applied, and a nominal frequency value (center of gravity of spectrum) was calculated for each pixel. The resultant image revealed a dependence on the underlying oxygen gradient, where a higher intensity corresponded to higher oscillation frequencies (Fig. 6b). A region near 12% oxygen showed brighter intensities than the surrounding concentrations. The data were replotted by averaging across the image to provide a modulation versus oxygen profile (Fig. 6c). In this profile, a peak frequency of 13.5 oscillations per hour was observed at 12% oxygen. This oscillation was equivalent to 4.4 min per cycle, which is comparable to reported values for whole islets and single beta cells at 4–5 min per cycle. This calcium modulation was consistent with the insulin jump and ATP dip that occurred in the same 10–12% region (Fig. 5). These data suggest that oxygen plays a key role for both the metabolic and electronic components in the dual oscillator model (Fig. 6d). Certain levels of oxygen represent a balance between these two components via ATP-related metabolites. We note that the overshoots of the calcium pulses were not included in this analysis, as they presented an asymmetrical artifact near 0% oxygen not seen in the adjacent corner (see Supplementary Data S3).

## Discussion

### Enhanced platform with gradient-based multimodal biosensing

We have created a platform for gradient-based multimodal biosensing enabling modulation of both oxygen and aqueous factors on beta cell cultures. The technique employed a custom rotational fluid shear apparatus for assessing gel adhesion on PDMS (Fig. 2b). Compared to conventional atomic force microscopy or mechanical shearing tests, the 3D printed device is easy to apply on stir plates and enables simple calculation of shear forces. The fluidic-based test is also compatible with cell-laden hydrogels and is applicable to numerous shear-dependent cellular studies. Using the multimodal gradient, we demonstrated spatial insulin sensing with a lower detection limit than conventional single point ELISA kits for 0-20% oxygen concentrations across an entire cell culture (Fig. 3d). The improved detection can be attributed to the hydrogel porosity and the direct path of insulin from the cell to the sensor. The platform also allows monitoring of calcium and mitochondrial ATP levels in situ, which revealed interesting dynamics at approximately 10% oxygen levels, as reported earlier^18,19^. Although the mechanisms of these dynamics are still unclear, our results suggest that they are related to changes in the levels of metabolites.

### Oxygen modulation of GSIR dynamics

By examining the spatiotemporal data from islets exposed to oxygen gradients, we observed a distinction between the effects on calcium transients versus the effects on insulin secretions. Unlike the optimal modulation of calcium transients (Fig. 6c), there was no optimal or peak insulin at the center of the gradient. Nevertheless, a discontinuity was seen at the same 12% oxygen level for insulin (Fig. 5c). We attribute the difference between insulin and calcium dynamics to the 2D, planar cell culture employed. For planar beta cells, glucose concentration is readily available and independent from specific oxygen concentrations. In 3D constructs and devascularized islets, both hypoxia and glucose diffusion overlap in a descending gradient toward the center of the microtissue. Our observation is yet another potential difference between 2D and 3D tissue structures.

Moreover, the observed oxygen effect appears to target GSIR oscillations. The current model of GSIR oscillations involves two components, as shown in Fig. 6d. In the slower metabolic component (3–6 min durations), phosphofructose kinase (PFK) feedback^23,28–30,40,41^ drives oscillatory mitochondrial ATP production. In the faster electrical component (10 s), membrane channel feedbacks modulate intracellular calcium flux^23,27,28,42–45^. A rise in intracellular calcium requires ATP pumps to recover, and ATP depletion then promotes PFK feedback in the metabolic component of the model^23,46^. As this dual oscillator model involves mitochondrial metabolism, oxygen consumption is also modulated^23,32,34^. Given this model, one potential mechanism of the observed oxygen effect is the limit on aerobic respiration, which shuts off the electron transport chain and promotes glycolysis and PFK feedback^33^. In comparison, our results showed that 8–12% hypoxia resulted in a decrease in mitochondrial ATP with a modest increase in the frequency of calcium oscillations. This is consistent with experiments in which a decrease in metabolic activity enhances cell networking and calcium oscillations via glycolytic effects on mitochondrial metabolism^36^. Furthermore, the dual oscillator model can also reproduce fast calcium oscillations when glycolysis is stationary^23,47^. The observed oxygen modulation of calcium could simply represent a dynamic balance between glycolysis and aerobic respiration. A second potential mechanism is the direct hypoxic modification of K_ATP_ channels and ion balance. This mechanism is observed in ischemia-reperfusion of tissue^48,49^ and our previous results in islet preconditioning^18^.

Another consideration is the difference in GSIR oscillations between whole islets versus single beta cells, where the latter is dominated by the slower metabolic component^23,43^. Although our cell culture conditions allow calcium and metabolites to distribute spatially across clusters of cells, we observed mostly slower oscillations related to the metabolic component of the model. The faster electrical component could simply be too weak and/or too sensitive^28,50^ to be observed by our minute-scaled spatial imaging compared to our faster fixed location, single oxygen results. Nevertheless, our data suggest that oxygen modulation of glucose-insulin oscillations occurs across the cell culture.

### Implications in studying islet pathogenesis and improving islet therapies

We note that there is a distinction between cell culture and physiological oxygen levels; atmospheric oxygen is 21%, whereas physiological blood oxygen is 10–13% (75–100 mmHg), and pancreatic tissue oxygen level is ~5.6% (40 mmHg)^51–54^. In earlier islet therapies, the hepatic portal vein was used as the transplant site (<2% O_2_ 5–15 mmHg), and those transplanted grafts suffered from hypoxia and shortened graft survival, which is associated with only 30-45% long-term insulin impendence^55–57^. Current research has been focused on improving islet hypoxia via hydrogel substrates, vascular grafts, novel transplant sites, and stem cell-derived islets. However, the specific effects of oxygen levels on GSIR are still unclear. Both our previous islet hypoxia study and the results in this work suggest a transitional oxygen level of 10–12%. This level of oxygen is higher than that in islets in vivo, and the difference may be due to the dense vascularization and convective transport of oxygen in the native pancreas versus the diffusion-limited transport in our cell culture devices. Nevertheless, the observed optimal oxygen level of 12% for calcium oscillation goes against the conventional view of a linear relationship between hypoxia and GSIR. Further studies on oxygen consumption rates, calcium flux, and intracellular metabolite levels could aid in interpreting this observed difference.

Additionally, future mechanistic studies could clarify the pathways affected by oxygen. Channel blocking or K_ATP_ knockout models can target the electronic oscillation pathway under different oxygen level conditions. Glucose starvation, oxygen consumption rates, and glycolysis tagging can target the metabolic oscillation pathway with different oxygen levels. Last, we have yet to explore the phase portion of our Fourier-based image analysis. This means that future studies comparing both pathways can be aided by phase maps to visualize the potential synchronization of adjacent beta cells, similar to what has been done in network analysis of GSIR^58–61^. Differential synchronization between faster electronic and slower metabolic oscillators can lead to a number of observed calcium response profiles^23^, with oxygen as a key controller in these processes.

In addition to the understanding of tissue hypoxia on islets, our results also suggest new strategies for improving islet therapies in the future. Our previous works leveraged microfluidics to improve islet selection for transplants. We have also demonstrated islet preconditioning to hypoxia^18^. Similarly, gradient modulation of calcium oscillation can precondition, or in this case, train naïve β-cells or stem cell-derived islets to synchronize their GSIR for a more responsive replacement tissue. This training strategy can be applied at oxygen levels determined per tissue sample and monitored by calcium imaging and network analysis. The goal would be to potentially improve the glucose sensitivity and magnitude of insulin secretion toward better islet therapy outcomes.

There is a limit in interpreting our cell culture results for native islet tissue. Beta cells in native islets are well vascularized and capable of cell-to-cell contact in all directions via gap junctions. Although our Beta TC6 cell culture also exhibits clusters that resemble tissue spheroids, synchronization of their calcium waves is probably inferior to that of the native islet construct. Nevertheless, this means native islets should see a more pronounced effect in the oxygen modulation of calcium oscillations, as we observed. It would be interesting and important to extend our oxygen modulation technique to whole islets for comparison in the future.

### Conclusion

This work was performed to create a robust multimodal gradient device to study β-cells under oxygen gradients. The realized microfluidic platform incorporated sensitive, spatial detection of insulin secretion in situ with an oxygen gradient. Using microfluidics, a peak calcium oscillation was observed at the 10–12% oxygen level, accompanied by a dip in mitochondrial ATP and a jump in insulin secretion. The evidence of an optimal oxygen level that is neither hypoxic nor normoxic counters the conventional view of a linear hypoxia relationship. Furthermore, the findings suggest oxygen to be a key parameter to balance the components within the dual oscillator model of GSIR but also raise further questions on the role that oxygen plays in either calcium channel modifications or metabolite modulations. The microfluidic technique enables experimental protocols to further answer these questions and provides a platform to apply training strategies for islet therapies. Furthermore, the technique for 3D fluid shear stress can provide a facile hydrogel adhesion test for biomaterial applications. Last, spatiotemporal analysis via time-based Fourier analysis can be applied to monitor growth factors, cytokines, and other secretory biomolecules to improve the visualization of molecular transport and gradients.

Supplementary Material: Figures S1 showing Beta TC6 cell viability, S2 showing ATP response compared to that of an INS1 cell line, and S3 showing data with overshoots are available as Supporting Information.

## Experimental section

### Fabrication of the microfluidic device

The device molds were fabricated using SU-8 photolithography methods. Briefly, an SU-8 2100 (Microchem) was spin-coated on a silicon wafer (University Wafer) to yield a 100 µm thick layer, soft baked and exposed to UV light through a mask. After the postexposure bake, it was finally developed with an SU-8 developer. The thickness of the channel for the gradient layer was measured using a surface profilometer. The surface of the wafer was plasma treated and then left in a vacuum overnight for salinization with trichloro (1H,1H,2H,2H-perfluorooctyl). To enable hydrogel bonding on top of the gas microfluidics, a double-sided PDMS membrane was fabricated using two SU8 masters First, a 200 µm membrane was molded over the gas microchannel master by spin coating PDMS. Then, a 100 µm membrane was molded on a separate wafer master to define the surface textures. These two membranes were bonded back-to-back to create a single layer with features of both sides. This two-sided membrane was then plasma bonded to a glass slide to enclose the gas channels with the textures exposed. Finally, a 1 cm thick PDMS gasket was cut and bonded to create the cell culture chamber.

### Polyethylene glycol diacrylate (PEGDA) hydrogel preparation

UV curable PEGDA hydrogel solution was prepared with the following: 8% PEGDA 6k (Sigma), 10% PEG 20k for porogen (Sigma), and 200 pg/mL of acrylated anti-insulin antibody. Anti-insulin was synthesized via the reaction between acrylate-PEG-SVA 5k (Laysan Bio) and anti-insulin (mouse MAB1 clone, Sigma) and incubated in phosphate-buffered saline (PBS) at room temperature for 3 h. Finally, 3 mM acrylamide-PEG5k-RGD and 2% photoinitiator 2-hydroxy-4′-(2-hydroxyethoxy)-2-methylpropiophenone (Sigma) were added to create the hydrogel precursor.

### PEGDA hydrogel on PDMS

Before pouring the hydrogel prepolymer solution onto the PDMS device, the textured PDMS surface was first plasma treated and then incubated in 5% acrylate-PEG-silane 10k (creating PEGWorks) for 2 h. Then, 100 µm PEGDA hydrogel solution was pipetted onto the PDMS and covered with a spacer to maintain a uniform gel thickness of 100 µm. The hydrogel solution was finally cured in a nitrogen chamber for 5 min. The entire procedure was conducted inside a nitrogen chamber to prevent PEGDA oxygen inhibition^62^. Finally, the device with the hydrogel was incubated with PBS for 5 h and washed several times to remove the porogen and create porosity in the hydrogel.

### Hydrogel bonding test

To test the adhesion of the hydrogel on PDMS, we designed a 3D printed support for the device. Then, we attached the device with the hydrogel facing down and placed the apparatus in a beaker full of water with a magnetic stir bar. The water was stirred at specified RPMs (rotations per minute) as verified by an optical tachometer. Membrane shearing was observed by eye, and the RPMs at which shearing occurred were recorded.

### Cell culture

Beta TC6 (ATCC) cells were cultured in high glucose Dulbecco’s modified Eagle’s medium (DMEM) (4500 mg/l glucose) with 15% heat inactivate fetal bovine serum (FBS) inside a cell culture incubator. To culture inside the microfluidics, 1.5 × 10^7^ Beta TC6 cells were loaded onto the device with 2 mL of medium without glucose. The device was incubated for 24 h prior to the start of experiments to allow Beta TC6 cells to reacclimatize to normal glucose levels before stimulation. During the microfluidic experiments, the media was replaced with Krebs–Ringer buffer (Boston Bioproducts) containing 129 mM sodium chloride, 5 mM sodium bicarbonate, 4.7 mM potassium chloride, 1.2 mM potassium phosphate monobasic, 1.2 mM calcium chloride, 1.2 mM magnesium sulfate, 1.2 mM heptahydrate and 10 mM HEPES.

### Oxygen gradient application

After 24 h of culturing, cells in the microfluidics device were washed with no glucose Krebs–Ringer and moved to an on-stage incubator on a microscope. Nitrogen and oxygen gas supplies were then connected to the device and adjusted to 18.30 and 7.14 sccm flow rates, respectively. The cells were incubated for 10 min to allow the gradient to stabilize, and then the experimental protocol described below was applied.

### Glucose stimulation

For glucose stimulation experiments, cells were then stained with 5 µM Fura-2AM for 10 min. The dye was replaced by no glucose Krebs–Ringer, and cells were then imaged for 10 min of incubation; then, cells were exposed to 1, 2, or 3 mM glucose-spiked Krebs–Ringer buffers and recorded for another 25 min. Finally, the cells were washed with buffer without glucose and recorded for another 10 more minutes.

### Insulin detection

The sensor optimizations were completed without cell culture in the device. After curing under UV light for 5 min, the PEGDA hydrogel was washed for 5 h to remove the porogen. The device was incubated with 3% BSA (Sigma) buffer to prevent protein absorption in PDMS and washed with PBS. Then, the sensor was incubated with different concentrations of insulin for various durations. After the insulin incubation, the device was washed with TBST buffer, and then the insulin reporter antibody (mouse anti-human with Alexa 594, R&D Systems) was incubated for different durations. For the optimization of insulin and reporter incubation times, the reporter antibody was diluted at 1:1000. The optimized incubation times were used for later experiments. For the optimization of anti-insulin in the hydrogel and detectivity assay, the reporter antibody was diluted 1:500. All subsequent cell-based experiments were performed with the same reporter antibody dilution.

### Mitochondrial ATP Imaging

ATP live cell fluorescence dye (BioTracker) was used to measure the ATP levels in the mitochondria of Beta TC6 cells. After 24 h of incubation in the microfluidic device, the glucose-free medium was removed and replaced with 2 mL Krebs-Ringer buffer containing 5 µM BioTracker dye for 15 min, followed by washing with no glucose Krebs–Ringer buffer. Cells in the device were imaged for 10 min. Then, the buffer was replaced by Krebs–Ringer with 3 mM glucose for stimulation and recorded for the next 25 min. Finally, the glucose buffer was replaced with buffer without glucose and recorded for another 10 min.

### Imaging

All images were taken using an Olympus IX75 microscope. All images were at 10× magnification. An approximately 7.5 × 1 mm slice of the symmetrical gradient was recorded for calcium, ATP, and insulin quantifications using the automated X/Y stage to generate image collages. Each slice contained 13,156 × 2556 pixels or 33 Megapixels. Forty-five timepoints at 1 min intervals were recorded before, during and after the glucose stimulation bolus.

### Calcium and insulin analysis

To calculate the fluorescence ratios for insulin detection, we used Olympus Cellsense software. An 8 × 10^5^ µm^2^ area was selected to calculate an average fluorescence intensity. The background image was measured by calculating the average intensity of the area without hydrogel. The ratio was then measured as the intensity divided by the background intensity.

The Fura ratio was calculated as the intensity of the 340 nm channel divided by the intensity of the 380 nm channel. The measurement process was the same for the insulin measurement. An 8 × 10^5^ µm^2^ area with cells was selected per measurement and tracked over the time points. The resultant time profile was leveled by removing the trend line as defined by the initial and end points.

### Temporal analysis via FFT

Processed Fura ratio images were imported into MATLAB. Twenty-five time points corresponding to the glucose stimulation pulses were cropped and used for the analysis. The initial time point was also removed to account for overshoot anomalies seen in Supplementary Data S3. The resultant image series contained 13,156 by 2556 pixels for each time point, resulting in a total of 33 Megapixel datasets. A per-pixel fast Fourier transform was applied across the time points. The absolute magnitude of the transform was used to calculate a nominal center of gravity frequency and replotted in the processed image. The processed image was normalized to show the frequency peaks seen at approximately 12% oxygen. Subsequently, the averaged frequency versus the gradient axis (13,156 pixel axis) was calculated and plotted as a summary of the 12% peak frequency.

### Live dead assay

Cell viability was determined by a Live and Dead Cell Assay (Abcam). After the cells were attached to the device for 0, 6, 12, and 24 h, they were washed with PBS, and 5× diluted live and dead dye in PBS was added to the device. After 10 min of incubation, the device was put under a fluorescence microscope for imaging.

### Statistical analysis

All the evaluations of statistical significance were performed using unpaired two-tailed Student’s *t* test; *p < 0.05, **p < 0.01, and ***p < 0.001. Figures with stars for statistical significance were plotted using the MATLAB Superbar function.

## Supplementary information

Supplementary Information

Supplementary figure 1

Supplementary figure 2

Supplementary figure 3(AB)

Supplementary figure 3(CD)
