# Supplementary material for: Visualizing hypoxic modulation of beta cell secretions via a sensor augmented oxygen gradient
Source: Microsyst Nanoeng. 2023 Feb 7;9:14. doi: 10.1038/s41378-022-00482-z (PMC9902275; doi:10.1038/s41378-022-00482-z)
Supplement: Supplementary file 1 — Supplementary Information [file 41378_2022_482_MOESM1_ESM.docx]

**Supporting Information**

**Visualizing hypoxic modulation of beta cell secretions via a sensor augmented oxygen gradient**

Kai Duan ^1^, Mengyang, Zhou, Yong Wang^2^, Jose Oberholzer^2^, Joe F. Lo^1^

1. Department of Mechanical Engineering, University of Michigan at Dearborn

2. Department of Surgery/Transplant, University of Virginia

We cultured Beta TC6 cells in 2D (surface coverage) on 100µm PEG porous hydrogel which was bonded to the PDMS membrane. RGD group co-polymerized in the hydrogel enabled the attaching of beta cells. Based on the result of culturing Beta TC6 cells in PEG porous hydrogel using this gradient microfluidic device, we found that after 24 hours culture, there were still more than 85 percent of cells alive, **S-1**.

To compare the GSIR of Beta TC6 to INS1 cells, we stimulated Beta TC6 at 3mM glucose and IN1 at 20 mM glucose and recorded their calcium and ATP responses. Beta TC6 calcium response is shown in **Figure 4A** of the main text. INS1 calcium response is shown in **S-2A**, with the expected pulse response over 25 minutes of stimulation, but without any obvious cycles or oscillations. Both Beta TC6 and INS1 cells were able to mount an ATP response to glucose at similar levels, **S-2B**.

During image process of oscillation analysis, an artefact was seen in the lower half near 0% oxygen that does not appear in the upper half, this was attributed to the overshoot component of the calcium transients. The overshoot of the calcium pulse was not included in this analysis in main article, the initial overshoot at 12 minute was removed to reduce artefacts, **S-3**.

**
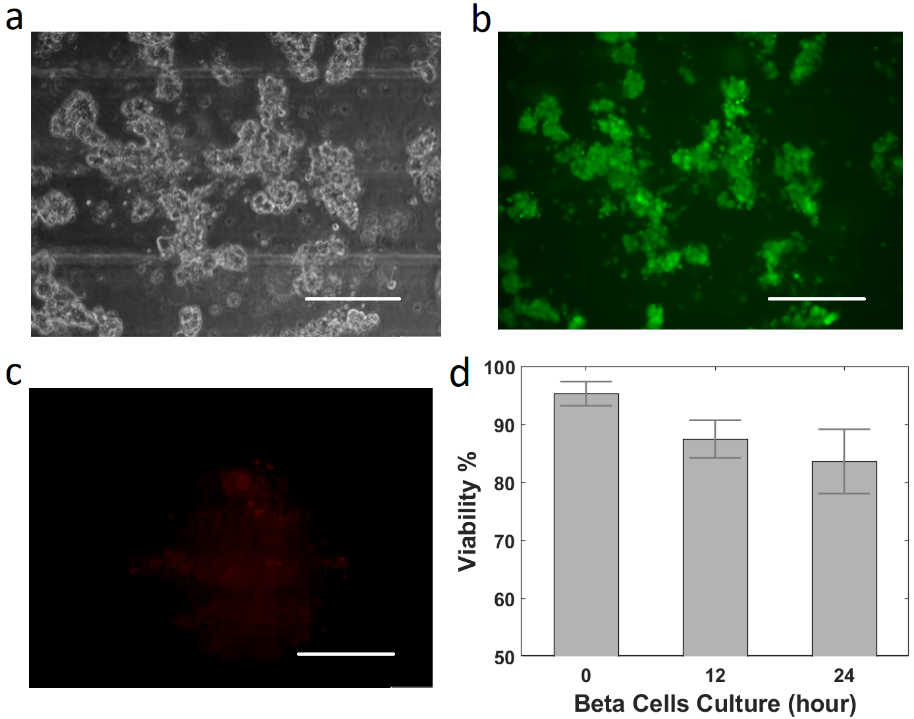
**

**S-1. Viability of Beta TC6 in gradient platform. a** Cells seeded on the gel laden PDMS surface. **b** Cleaved calcein fluorescence showing attached cells. Cells also appear in clusters that promotes cell-cell interactions. **c** Ethidium homodimer fluorescence showing isolated dead cells. (scale bar denotes 200 µm). **d** Compiled viability data for Beta TC6 under 24-hour cell culture with better than 85% viability.


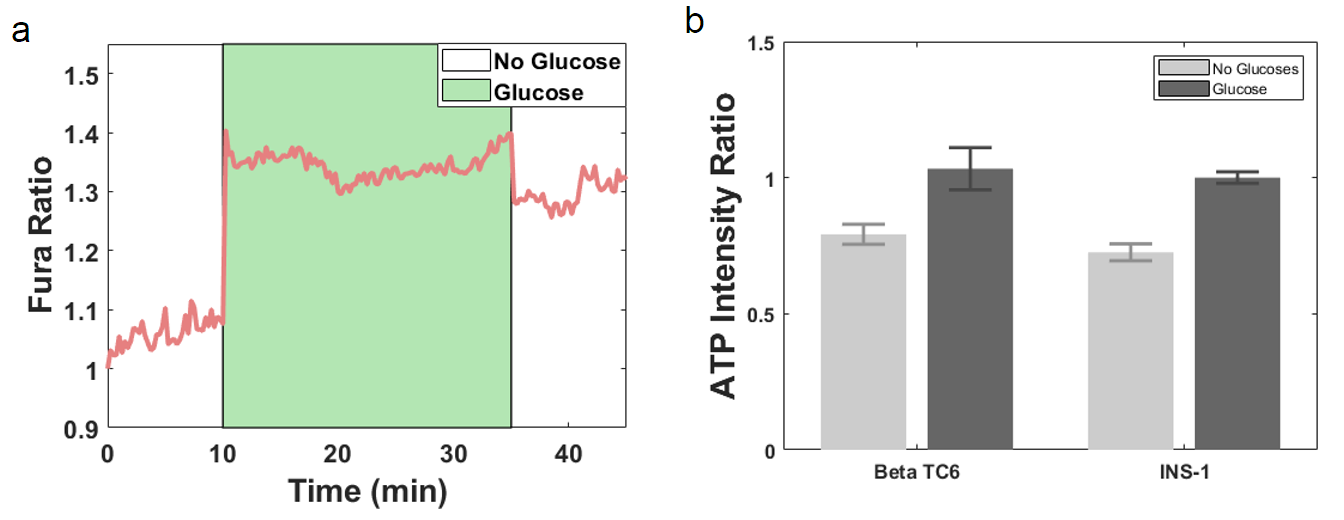


**S-2. Beta TC6 and INS1 cells had similar ATP response to glucose. a** INS1 cells had calcium response to 20 mM glucose without any obvious oscillations, compared to the oscillations seen in Beta TC6 shown in the main text. **b** Both cell lines produced an ATP response to glucose, with similar levels over baseline.


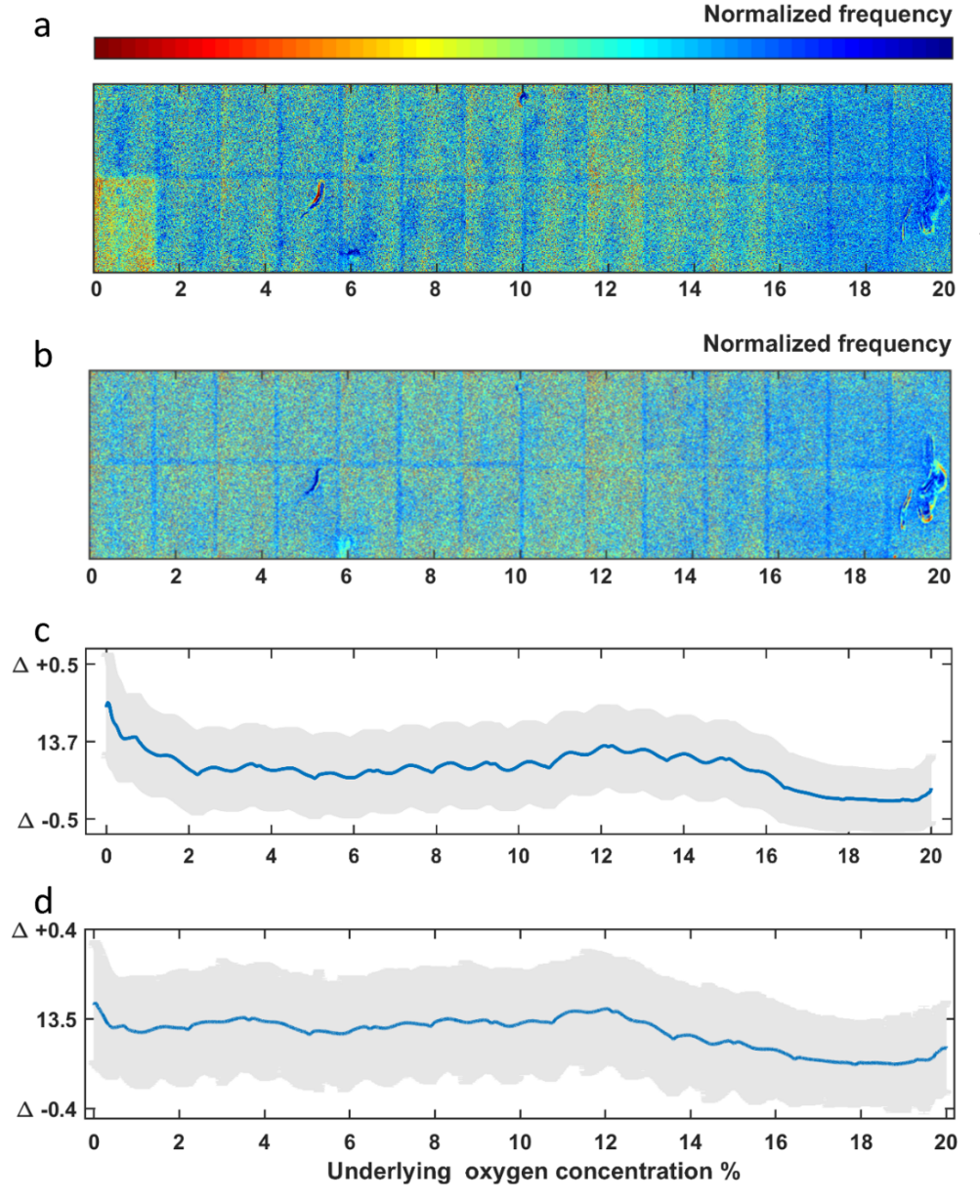


**S-3. Comparison of image processing with and without initial overshoot. a** Processed image including 12-36 minutes time points for the entire 3 mM glucose bolus. An artefact was seen in the lower half near 0% oxygen that does not appear in the upper half. This was attributed to the overshoot component of the calcium transients. **b** When the overshoot at 12 minutes time point was removed, the artefact was also removed, showing a symmetric image top to bottom of the collage. **c** The collapsed oscillation versus oxygen profile, showing strong artefact near 0% oxygen. **d** When the overshoot was removed, the artefact was minimized and the peak around 12% then came into sharper relief.
